# Drug-resistant tuberculosis control in China: progress and challenges

**DOI:** 10.1186/s40249-016-0103-3

**Published:** 2016-01-29

**Authors:** Qian Long, Yan Qu, Henry Lucas

**Affiliations:** Global Health Research Center, Duke Kunshan University, Kunshan, China; Duke Global Health Institute, Duke University, Durham, NC USA; China Center for Disease Control and Prevention, Beijing, China; Institute of Development Studies, Sussex University, Brighton, UK

**Keywords:** Multidrug-resistant tuberculosis, Health financing, Health system strengthening, China

## Abstract

**Background:**

China has the second highest caseload of multidrug-resistant tuberculosis (MDR-TB) in the world. In 2009, the Chinese government agreed to draw up a plan for MDR-TB prevention and control in the context of a comprehensive health system reform launched in the same year.

**Discussion:**

China is facing high prevalence rates of drug-resistant TB and MDR-TB. MDR-TB disproportionally affects the poor rural population and the highest rates are in less developed regions largely due to interrupted and/or inappropriate TB treatment. Most households with an affected member suffer a heavy financial burden because of a combination of treatment and other related costs. The influential Global Fund programme for MDR-TB control in China provides technical and financial support for MDR-TB diagnosis and treatment. However, this programme has a fixed timeline and cannot provide a long term solution. In 2009, the Bill and Melinda Gates Foundation, in cooperation with the National Health and Family Planning Commission of China, started to develop innovative approaches to TB/MDR-TB management and case-based payment mechanisms for treatment, alongside increased health insurance benefits for patients, in order to contain medical costs and reduce financial barriers to treatment. Although these efforts appear to be in the right direction, they may not be sufficient unless (a) domestic sources are mobilized to raise funding for TB/MDR-TB prevention and control and (b) appropriate incentives are given to both health facilities and their care providers.

**Summary:**

Along with the on-going Chinese health system reform, sustained government financing and social health protection schemes will be critical to ensure universal access to appropriate TB treatment in order to reduce risk of developing MDR-TB and systematic MDR-TB treatment and management.

**Electronic supplementary material:**

The online version of this article (doi:10.1186/s40249-016-0103-3) contains supplementary material, which is available to authorized users.

## Multilingual abstracts

Please see Additional file 1 for translations of the abstract into the six official working languages of the United Nations.

## Background

After two decades of international efforts to combat tuberculosis (TB), the global mortality rate (deaths per 100,000 population per year) has decreased by 45 % since 1990 and incidence rates (new cases per 100,000 population per year) are also falling in most parts of the world [1]. However, the increasing prevalence of drug-resistant tuberculosis (DR-TB) is undermining global TB control. In 2012, there were an estimated 450,000 patients suffering from multidrug-resistant tuberculosis (MDR-TB) (defined as tuberculosis caused by strains of Mycobacterium tuberculosis that are at least resistant to rifampicin and isoniazid treatment), and an estimated 170,000 deaths from MDR-TB [1]. It was also estimated that more than 75 % of those with MDR-TB were not diagnosed, the majority in countries with a high MDR-TB burden [1]. This situation is generally ascribed to a combination of insufficient laboratory facilities, a lack of appropriately qualified health professionals and weak TB surveillance systems. Globally, around 82 % of detected MDR-TB patients in 2012 had started second-line treatment. However, treatment coverage varied notably by geographical area, with only 51 % of MDR-TB patients under treatment in the World Health Organization (WHO) African Region in that year [1]. Undetected MDR-TB cases and treatment coverage gaps have been highlighted as constituting a global public health crisis in the 2013 Global TB Report.

The WHO recommends a TB control strategy that includes government commitment, early diagnosis by bacteriologic testing, standardized treatment and regular patient management. This DOTS (directly observed treatment, short-course) strategy [2] has made notable progress in global TB control, saving an estimated 6 million lives between 1995 and 2008 [3]. In 2005, the Stop TB Partnership launched a new strategy for 2006–2015, which specified financing and management interventions for both drug-susceptible and drug-resistant TB control. However, it has been argued that both funding and implementation have lagged far behind the action plan targets, and that political commitment on MDR-TB prevention and control is insufficient [4]. In 2009, WHO, the Bill and Melinda Gates Foundation and the Chinese Ministry of Health (now called the National Health and Family Planning Commission of China, NHFPC) organized a ministerial meeting in Beijing calling for action to tackle TB and DR-TB in 27 countries with a high burden of MDR-TB and extensively drug-resistant tuberculosis (XDR-TB) [5]. In the meeting, China reported a serious MDR-TB epidemic and agreed to draw up a plan for MDR-TB prevention and control in the context of a comprehensive health system reform launched in the same year.

The new round of health system reforms in China has emphasized the role of government in funding and supervision, and aims to achieving equitable and affordable access to quality health care for all. Over 2009–2011, the Chinese government committed to invest CNY 850 billion (US$ 125 billion) and set five key reform priorities: 1) speeding up the establishment of three basic health insurance schemes (the rural New Cooperative Medical Scheme (NCMS), the Urban Residence Basic Medical Insurance (URBMI), and the Urban Employee Basic Medical Insurance (UEBMI)) and medical financial assistance for the poor; 2) developing a national system to ensure the availability and affordability of quality essential medicines for all; 3) strengthening primary health care facilities, particularly in the rural areas and urban community settings to ensure the provision of cost-effective care; 4) promoting and improving equity in public health services; 5) exploring public hospital reform through pilot studies [6]. The achievements over these three years are impressive, including universal health insurance coverage, improved infrastructure of primary health facilities and increased uptake of services [7].

The reform has recently entered a second phase to tackle remaining challenges. The new four-year plan (2012–2015) focuses on unifying China’s three health insurance schemes and increasing benefits, encouraging payment reform to avoid perverse financial incentives to healthcare providers, introducing performance-based funding for providers, expanding community and public health services, and regulating drug production, prescription and pricing [8]. The government commitment to health system reform remains strong and the process is followed keenly by national and international stakeholders.

To tackle TB, and particularly the MDR-TB epidemic, strengthening the health system is critical. This paper reviews the burden of MDR-TB and factors driving MDR-TB in China, and then discusses the development of MDR-TB prevention and control in line with the on-going Chinese health system reform with a focus on financing for TB/MDR-TB care.

## Discussion

China has the second highest caseload of MDR-TB in the world [1]. According to the National Survey of Drug-Resistant TB in 2007, one third of new TB cases and one half of previously treated cases exhibited anti-TB drug resistance. Some 5.7 % (range 4.6–7.1 %) of new cases and 25.6 % (range 21.7–30.0 %) of previously treated cases developed MDR-TB [9]. The results of this survey confirmed an estimate of around 100,000 emerging MDR-TB cases annually in China. Among MDR-TB patients, 7.2 % (range 4.9–10.2 %) were diagnosed as XDR-TB, around 8,200 cases [9]. In addition, 11 % of new cases and 16 % of previously treated cases were resistant to either isoniazid or rifampicin and were at high risk of developing MDR-TB [10]. Likewise, one third of patients with MDR-TB had drug resistance to either ofloxacin or kanamycin [10] and were one step away from XDR-TB. These findings have sounded an alarm that the prevalence of MDR-TB and XDR-TB in China could easily increase.

Although knowledge gaps as to the causes of MDR-TB remain, interrupted and/or inappropriate TB treatments have been identified as the most important contributory factors in China [10–12]. It has been argued that they can be at least partially attributed to health system failures, in particular the reliance on a fee-for-services approach to financing public health facilities. TB has been seen as a disease of the poor. It is not surprising that the risk of developing MDR-TB is highest for poor and/or vulnerable members of the population. Most cases are found in the less developed northeast and southwest regions [13]. Some 80 % of MDR-TB patients are from rural areas, and most had low education and were in the young to middle age-groups [14].

In the 1990s, the national TB control programme required the prompt referral of TB suspects who had a cough for more than three weeks, hemoptysis or blood in a sputum sample to TB dispensaries for diagnosis, and provided free first-line anti-TB drugs for diagnosed patients. This was seen as essential in order to remove, or at least substantially decrease, financial barriers to accessing standard TB care by the poor. However, according to an evaluation of China TB control in 2004–2005, 70 % of suspects were not referred to TB dispensaries for diagnosis [15] but treated as non-TB cases in public hospitals. Most patients spent more than half of their annual income on treatment before being diagnosed with TB [16]. In addition, many studies in China have reported that TB patients are charged for longer treatment periods than recommended by the TB control programme and that drugs and tests are administered to an extent considerably beyond that specified in the standard treatment regimen [17, 18]. As a result, many patients have paid excessive fees for TB treatment or have dropped out and/or discontinued treatment because of difficulties in affording care [19]. In addition, a lack of proper training has often resulted in irrational prescriptions and treatments [12].

Treatment of MDR-TB is complicated, expensive and often unsuccessful, resulting in a low cure rate, high mortality rate and low follow-up rate [20]. The WHO guidelines for MDR-TB recommend 18–24 months of chemotherapy using a combination of first- and second-line drugs (including daily injections in the first 6–8 months). One systematic review that identified four studies on the cost of MDR-TB treatment found that the cost per case was substantially higher in two locations where routine care included substantial hospitalization (US$ 14,657 in Tomsk and US$ 10,880 in Estonia) than where it only involved ambulatory care (US$ 3,613 in the Philippines and US$ 2,423 in Peru) [21]. One study in China reported average daily medical costs for MDR-TB treatment were more than three times the average cost of household daily non-food consumption in Tianjin city and one and a half times in Henan province [22]. Some 92 % of MDR-TB patients in Tianjin and 70 % of patients in Henan experienced catastrophic healthcare payments (defined in this study as daily medical costs being over 40 % of daily non-food consumption) [22].

### Progress on MDR-TB control in China

Drug-resistant TB prevention and control, especially with regards to MDR-TB, has been an important component of the national TB control programme in China [19]. Since the 1990s, China has adopted the DOTS strategy for systematic management of TB cases, implemented in a semi-vertical TB control system affiliated with the Center for Disease Control and Prevention (CDC) at four levels: national, provincial, prefectural, and county/district. National and provincial TB prevention and control centers were usually responsible for programme administration, TB care supervision and case reporting. Lower level TB dispensaries focused on the diagnosis of TB suspects, treatment and management. TB patients with complications were referred to public hospitals. In 1998, with the increasing prevalence of MDR-TB and other TB related diseases (e.g., TB/HIV, TB/diabetes co-infections etc.), pilot exercises to integrate TB care into public hospitals was undertaken in Shanghai, Jiangsu and Zhejiang provinces and a few sites in the less developed western areas. TB clinics were set up within public hospitals to provide TB/MDR-TB diagnosis and treatment and to cooperate with the local TB dispensary on case management and reporting. This so called ‘designated hospital model’ for TB control [23] is now being scaled up.

The central government provided annual earmarked funding to cover the costs of first-line anti-TB drugs, two X-ray examinations and five sputum smear tests for TB, but there was no designated fund for MDR-TB diagnosis and treatment at the national level [24]. Several international donors supported targeted interventions on MDR-TB prevention and control in China, which were often project/programme based. For example, over 2010–2014 the Global Fund supported a programme on strengthening MDR-TB management that was implemented in 89 prefectures of 30 provinces [25]. The main contents of this programme included: drug susceptibility testing (DST) with smear-positive patients; DR-TB surveillance in project sites; the introduction of rapid MDR-TB diagnosis technology; covering the cost of hospital admission for MDR-TB treatment; providing MDR-TB patients with a transport subsidy; improving the quality of second-line drugs produced in China; and ensuring a consistent supply of second-line drugs. Matching funds from both provincial and prefecture levels were required to co-financing the programme [25]. In the project sites of the Global Fund, 62 % of registered TB cases were tested for drug susceptibility in 2010. The ratio of MDR-TB cases diagnosed to enrolments on MDR-TB treatment was 57 % and over two thirds of MDR-TB patient sputum culture examinations were negative by the end of 6-months of treatment, which was much higher than in non-project sites [26]. However, the Global Fund programme is time-limited and sustainable long-term interventions need to be established.

China’s ongoing health system reform towards universal healthcare coverage provides important opportunities to improve access to appropriate care to prevent, diagnose and treat TB/MDR-TB and protect patients from financial hardship. In 2009, a comprehensive programme that aims to improve MDR-TB diagnosis and the quality and affordability of treatment was developed and implemented in four cities, supported by the Government of China and the Melinda and Bill Gates Foundation. The strategies included: the introduction of rapid molecular diagnosis for isoniazid and rifampicin susceptibility and associated laboratory staff training; standardized and detailed MDR-TB treatment protocols based on the estimated degree of drug resistance; the use of health insurance and other funds to cover 90 % of the medical costs of MDR-TB diagnosis and treatment and to subsidize transport and nutritional supplementation; and strengthening MDR-TB patient management using a TB control network, particularly in primary care facilities and the community. The effects of the programme have been impressive, with a substantial increase in the number of diagnosed patients and use of appropriate treatment. There has also been a significant decrease in the average ratio of out-of-pocket payment to annual household income [27].

In 2012 China CDC and the Foundation initiated Phase II of the programme. In addition to confirmed effective diagnosis, treatment and patient management approaches, this emphasizes a sustainable financing mechanism for TB/MDR-TB treatment. It was proposed that health insurance schemes (NCMS, URBMI and UEBMI) should cover both inpatient and outpatient TB care and that the reimbursement rate should be increased to 80 % for TB treatment and 90 % for MDR-TB treatment. This was intended to reduce the financial burden on TB/MDR-TB patients and improve patient adherence to treatment. A case-based payment mechanism for TB/MDR-TB treatment was designed to ensure cost containment and standardized clinical practice. In addition, the provision of a transport subsidy for TB/MDR-TB patients was explored through cooperation with the Civil Affairs Bureau which is in charge of the Medical Financial Assistance scheme. The strategies for MDR-TB control in this programme are shown in Table 1. Health financing and payment reform is a critical component of the overall Chinese health system reforms. Good practice and lessons drawn from this ongoing TB control programme will be valuable for the ongoing development of health financing policy.

**Table 1:** Strategies for MDR-TB control in the China-Gates TB programme Phase II

Technical approach	System strengthening	Financing and payment
**•** MDR-TB detection: All smear or culture positive patients are tested for drug susceptibility using rapid molecular test	**•** Referral: County TB designated hospitals are required to refer all MDR-TB patients (Rifampicin resistant inclusive) to city TB designated hospital for diagnosis and treatment	**•** Health insurance benefits: The reimbursement rate should be 90 % for MDR-TB treatment
**•** MDR-TB treatment: All MDR-TB (Rifampicin resistant inclusive) patients are treated with standard second line TB regimen and tailed regimen based on tested degree of drugs resistance	**•** Patient management: 1) Staffs in local CDC and primary health facilities are responsible for MDR-TB patients management for 24 months; 2) MDR-TB patients management information shared between TB designated hospitals and CDCs without barriers	**•** Transportation subsidy for patients through grant from local Civil Affairs Bureau
		**•** Case-based payment for the full course of MDR-TB treatment

Data source: Baseline survey report of the comprehensive TB control model, China CDC, 2013

### Challenges on MDR-TB control in China and the way forward

Efforts to combat TB/MDR-TB in China have made substantial progress. However, critical weaknesses could jeopardize effective implementation of the current strategy. In this paper, we discuss challenges to TB/MDR-TB control in China from a health financing perspective.

Although government funding for TB/MDR-TB control has increased year by year, there remains a substantial shortfall in terms of providing the financial support needed to ensure effective TB/MDR-TB diagnosis, treatment and management. Based on data from the national population census in 2010, and the national TB and MDR-TB surveys in 2010 and 2007 respectively, an estimated CNY 13 million per annum is required for TB control in each prefecture and CNY 4.6 million for MDR-TB [28]. A survey carried out in three prefectures in 2013 found that the annual funding allocated to TB control was only one third of that required in two prefectures in the eastern and central regions and much less in the western prefecture [24]. Funding for MDR-TB was far below the suggested level in all three prefectures, and largely relied on the Global Fund, even in the developed eastern region [24]. When the Global Fund programme for MDR-TB control ends, the funding shortfall for MDR-TB will increase substantially if domestic sources are not mobilized.

It is of great concern that the less developed regions often have a higher burden of TB/MDR-TB but proportionally much lower expenditure on prevention and control. This reflects the limited funds allocated to health services in general, which can in turn be linked to their overall poor fiscal status. With the decline in international donor funding, it has been proposed that low- and middle-income countries, especially the emerging economies (e.g., Brazil, Russia, India, China and South Africa) should increase the amount they spend on combating the MDR-TB epidemic as a means of ensuring sustainable development. China’s economic success over the reform period has generated the resources which would allow a substantial increase in the funding of initiatives to address the MDR-TB public health crisis. These initiatives will need to take into account regional disparities in both the challenges faced by those confronting TB/MDR-TB and the overall financial resources available at the local level.

In China, MDR-TB diagnosis and treatment practices vary by prefecture. In general, TB designated hospitals and the CDC at city and/or higher levels are able to conduct DST and MDR-TB treatment. In some settings, county or district TB designated hospitals and/or the CDC are responsible for delivering the samples from all smear-positive patients and treatment failure patients to city or higher level facilities for sputum culture examinations and DST. In some settings, smear-positive and treatment failure patients are recommended to visit city or higher level facilities in person for DST.

According to the CDC TB register, the average delay in 2013 between identification as an MDR-TB suspect and an MDR-TB diagnosis ranged from 59 to 83 days in the three prefectures in the eastern, central and western regions identified above [24]. Interviews with the head of the TB department in the local CDC, TB designated hospital managers and TB care providers in each of the three prefectures identified some common constraints resulting in long delays in MDR-TB diagnosis. These included lack of funds and/or incentives at county or district TB facilities to ensure the correct and timely delivery of patient samples, and the perception by patients, especially poor patients, that they would have difficulty in affording care [24].

After diagnosis, it is recommended that all MDR-TB patients should be admitted to a prefecture TB designated hospital for an initial 1–2 month period and then should continue outpatient treatment for 16–18 months. Treatment is individualized, being determined by the specific drugs to which a patient exhibits resistance, and the cost is some ten times that of standard TB treatment [28]. Although most rural patients have NCMS coverage, the reimbursement rate for hospital admission is usually low (based on the NCMS principle of lower reimbursement rates at higher level health facilities) and outpatient costs are often not covered. A survey in three prefectures located in the eastern, central and western regions in 2013 reported that the average out-of-pocket payment for MDR-TB treatment over a 24 month period was CNY 20,544 [24]. Non-medical costs (including fees for transport and accommodation during treatment) were around one third of the medical cost. In this survey, almost all patients reported that the financial burden on their households was heavy or very heavy and half reported borrowing money from their relatives or a bank to pay for treatment. These three prefectures were all sites covered by the Global Fund programme, which provided financial support for treatment and transport. It may be inferred that MDR-TB treatment would be less affordable in non-project sites and might give rise to more serious economic and social consequences.

The China-Gates project Phase II introduced new financing and payment methods to reduce the financial barriers to accessing MDR-TB treatment (including cooperation with the Civil Affairs Bureau to subsidise patient travel costs). However, it was recognized that without appropriate incentives to both TB designated hospitals and their TB care providers, there was a risk of undermining this intervention. At the prefecture level, the revenues of TB designated hospitals were still largely from service charges [24]. In qualitative interviews with prefecture health administrators, CDC directors and TB designated hospital managers in the three prefectures, most expressed the opinion that the implementation of case-based payments for TB/MDR-TB treatment in prefecture TB designated hospitals would lead to a decline in hospital revenues [24]. This would reduce the incentive to treat TB/MDR-TB and might negatively impact the quantity and/or quality of care provided. Another important consideration is that the salaries of TB care providers in hospitals are directly related to the associated service fees. Most TB care providers interviewed were not satisfied with their current salaries, which were typically less than their colleagues in other departments. There was also concern that they might be exposed to a high occupational risk in treating infectious diseases. This had made it difficult to recruit TB doctors [24].

Financial and material incentives have been directed at individual healthcare providers and organizations (both public and private) in many other low- and middle-income countries, aiming to improve the quality of TB diagnosis and treatment, typically alongside system changes intended to promote improved outcomes. For example, in Romania and Honduras incentives (e.g., gift tickets or other materials) were given to public healthcare providers conditional on objective performance indicators, for example the number of new cases confirmed by microscopy, the rate of DOT in sputum-positive patients, and patient attendance for TB treatment [29]. In India, the Philippines and Myanmar, private healthcare providers were supplied free anti-TB drugs on condition that patients did not pay for these [29, 30]. Dispensing free drugs is seen as an incentive for private providers because they can charge consultation fees and develop their reputation for curing TB patients, which might raise client demand for other services. There have also been trials involving the provision of conditional financial incentives at organization level (e.g., non-governmental organizations, anti-TB teams, local governments) linked to TB control performance indicators [29]. It is difficult to fully attribute performance changes to these incentives given that they were often just one component of multifaceted interventions. However the evidence does seem to indicate increased effectiveness in case detection and treatment completion where incentives were offered to providers and/or patients [29]. Findings from studies in a number of countries emphasise the need for great care in both the design of such incentives and their implementation, which requires a detailed understanding of the environment within which providers operate and their needs, as well as scientific evaluation of effectiveness. These international experiences should be considered in the on-going reform of TB diagnosis and treatment financing in China. Developing a hospital compensation strategy that includes appropriate incentives for TB care providers will be an essential component of an effective intervention.

## Summary

The high prevalence of drug-resistant TB, especially MDR-TB and XDR-TB, is a global public health crisis. In 2009, the Beijing Call for Action and the World Health Assembly Resolution 62.15 made clear that the actions taken to address this crisis by national TB control programmes were insufficient. Radical policy changes were required which would involve the strengthening of health systems and services and increased government accountability [31, 32].

The Chinese government commitment on combating TB/MDR-TB remains strong. In the context of a new round of Chinese health system reforms, the Bill and Melinda Gates Foundation in cooperation with the government of China started to develop innovative approaches to TB/MDR-TB prevention and control including 1) new MDR-TB diagnosis, treatment and management mechanisms; 2) increased health insurance benefits and a travel subsidy provided by the Civil Affair Bureau to remove financial barriers to access to treatment; 3) a case-based payment method to contain costs. Although these efforts appear to be in the right direction, they may not be sufficient unless: 1) domestic sources are mobilized to raise funding for TB/MDR-TB prevention and control; and 2) appropriate incentives are given to both health facilities and their care providers. Overall, the policies required to achieve these objectives cannot be limited to the health sector but will need cooperation across sectors including finance, social welfare and labour. Findings from the on-going China-Gates TB programme Phase II will require careful analysis and interpretation in relation to the effects of TB/MDR-TB control and financial protection for patients in order to guide evidence-based policy development.

## Additional file

Additional file 1:
**Multilingual abstracts in the six official working languages of the United Nations.** (PDF 365 kb)

## Abbreviations

DOTS: Directly Observed Treatment, Short-course
DR-TB: Drug-Resistant Tuberculosis
DST: Drug Susceptibility Testing
MDR-TB: MultiDrug-Resistant Tuberculosis
NCMS: New Cooperative Medical Scheme
NHFPC: National Health and Family Planning Commission of China
TB: Tuberculosis
UEBMI: Urban Employee Basic Medical Insurance
URBMI: Urban Residence Basic Medical Insurance
WHO: World Health Organization
XDR-TB: Extensively Drug-Resistant Tuberculosis
