# Supplementary material for: Drug-resistant tuberculosis control in China: progress and challenges
Source: Infect Dis Poverty. 2016 Jan 29;5:9. doi: 10.1186/s40249-016-0103-3 (PMC4731907; doi:10.1186/s40249-016-0103-3)
Supplement: Additional file 1: — Multilingual abstracts in the six official working languages of the United Nations. (PDF 365 kb) [file 40249_2016_103_MOESM1_ESM.pdf]

Translation of the abstract into the six official working languages of the United Nations

جهود السيطرة على داء السل المُعنّد على الدواء في الصين: الإنجازات والتحديات

كيان لونغ ، يان كو ، هنري لوكاس

ملخص

معلومات تمهيدية: تُعد الصين الدولة الثانية في العالم من حيث عدد حالات الإصابة بداء السل المُعنّد على العديد من الأدوية أدوية المتعددة السل المقاوم (السل المقاوم للأدوية المتعددة). وافقت الحكومة الصينية في العام 2009 على رسم خطة للوقاية من داء السل المُعنّد على العديد من الأدوية والسيطرة عليه وذلك في سياق خطة شاملة للإصلاح الصحي انطلقت في نفس العام.

نقاش: تواجه الصين معدلاتٍ انتشار كبيرة لداء السل المُعنّد على الدواء السل المقاوم للأدوية وداء السل المُعند على العديد من الأدوية السل المقاوم للأدوية المتعددة . يُصيب داء السل المُعند على العديد من الأدوية المناطق الفقيرة والريفية على نحوٍ غير متكافئ، ويكون الانتشار الأكبر لهذا المرض في المناطق الأقل تحضراً بسبب العلاج غير الكامل أو غير المناسب لداء السل. تواجه الأسر التي يعاني أحد أفرادها من الإصابة بالسل من أعباء مالية كبيرة بسبب التكاليف الباهظة للعلاج والرعاية الصحية للمريض. يُوفّر برنامج الصندوق العالمي للسيطرة على داء السل المُعنّد على العديد من الأدوية في الصين برنامج الصندوق العالمي لمكافحة السل المقاوم للأدوية المتعددة في الصين دعماً تقنياً ومالياً لتشخيص وعلاج هذا المرض. ولكن الجدول الزمني لهذا البرنامج مُحدد وثابت، ولا يمكن أن يُقدم حلاً طويل الأمد للمشكلة. في العام 2009 بدأت مؤسسة بل وميلندا جيتس مؤسسة بيل وميليندا غيتس بالتعاون مع الهيئة الوطنية للصحة وتنظيم الأسرة في الصين بتطوير طريقة مبتكرة لعلاج داء السل وداء السل المُعند على العديد من الأدوية، وآليات لتسديد التكاليف العلاجية بناءً على كل حالة، وذلك بالتوازي مع زيادة مستحقات التأمين الصحي للمرضى كي يصبح من الممكن تغطية النفقات العلاجية للمرض، وتذليل العقبات المالية التي تحول دون حصول المرضى على العلاج. وعلى الرغم من أن هذه الجهود تصب في الاتجاه الصحيح، إلا أنها تظل غير كافية ما لم يتوفر عاملين: 1- مصادر محلية لتغطية تكاليف الوقاية والعلاج من داء السل وداء السل المُعند على العديد من الأدوية، 2- حوافز مناسبة لكل من المؤسسات الصحية ومزوّدي الرعاية الصحية الذين يعملون فيها.

الخُلاصة: بالتوازي مع خطة إصلاح القطاع الصحي في الصين، لا بد من توفير مصادر تمويل حكومية مستمرّة وحلول ضمان صحي اجتماعي للتأكد من حصول جميع المرضى على العلاج المناسب للسل، وبالتالي الحد من خطر الإصابة بداء السل المُعند على العديد من الأدوية، وعلاج وتدبير السل المُعند على العديد من الأدوية بشكل منهجي.

Translated from English version into Arabic by Sari M. Barazi, through

TRANSLATORS
WITHOUT BORDERS

中国耐药结核防控：进展与挑战

龙倩，屈燕, Henry Lucas

背景：中国是耐多药结核高负担国家之一。2009 年，中国政府决定与新一轮中国医疗卫生体制改革相结合，制定耐多药结核的预防和控制策略。

讨论：中国正面临着较严重的耐药结核与耐多药结核的流行。在欠发达地区和贫困的农村人口中，耐多药结核患病率最高。而耐多药结核的发生，主要是由间断的或不恰当的结核治疗造成的。由于耐多药结核治疗时间长，其医疗和其他相关的费用通常对耐多药结核病人的家庭带来沉重的经济负担。中国全球基金耐多药结核病控制项目，对耐多药结核诊断和治疗提供技术和经费上的支持。然而，这样的项目有其项目期限，并不是长久之计。自 2009 年，比尔及梅琳达盖茨基金会与中国国家卫生和计划生育委员会合作，发起和实施新型结核/耐多药结核的诊断，治疗，病人管理办法以及新的筹资与支付办法，如实施单病种付费，

同时增加医保的报销比例，以便控制医疗费用，减轻病人的经济负担。然而，欲确保新策略的成功实施，仍需加大国家对结核，尤其是耐多药结核的投入，以及提供恰当的对医疗机构和结核医护人员的激励。
小结： 随着新一轮中国医疗卫生体制改革的推进，持续的政府投入和社会保障与医疗保障体系的逐渐完善和成熟，是促进结核诊疗公平性的关键，进而减少发展耐多药结核的风险，并确保耐多药结核病人能接受系统的耐多药结核的诊疗和管理。

Translated from English version into Chinese by Long Qian

Lutte contre la tuberculose résistante en Chine : avancées et difficultés

Qian Long, Yan Qu, Henry Lucas

Résumé
Contexte : La Chine est le deuxième pays au monde par le nombre de cas de tuberculose multirésistante (MR). En 2009, le gouvernement chinois s’est engagé à mettre en place un plan de prévention et de lutte contre la tuberculose MR dans le cadre d’une vaste réforme du système de santé publique lancée la même année.
Discussion : La prévalence de la tuberculose résistante et multirésistante est élevée en Chine. Elle affecte en particulier les populations rurales défavorisés, et les taux de prévalence plus élevés dans les régions les moins développées s’expliquent en grande partie par le nombre de traitements antituberculeux interrompus et/ou inappropriés. La plupart des foyers dont un membre est atteint doivent supporter une lourde charge financière à cause du traitement et des autres frais connexes. L’influent programme mis en place par le Fonds Mondial pour lutter contre la tuberculose MR en Chine apporte un soutien technique et financier pour le diagnostic et le traitement de cette forme résistante de la maladie. Il est toutefois limité dans le temps et ne peut constituer une solution à long terme. En 2009, la Fondation Bill et Melinda Gates, en coopération avec la Commission nationale de la santé et du planning familial chinoise, a commencé à étudier des approches innovantes pour lutter contre la tuberculose, y compris multirésistante, et des mécanismes de paiement par cas pour son traitement, ainsi qu’une augmentation des prestations d’assurance maladie pour les patients, afin de maîtriser les frais médicaux et de réduire les obstacles financiers au traitement. Bien que ces efforts semblent aller dans le bon sens, ils ne seront peut-être pas suffisants, à moins (a) que des ressources intérieures soient mobilisées pour financer la prévention et la lutte contre la tuberculose (et plus spécifiquement la tuberculose MR) et (b) que des incitations appropriées soient proposées à la fois aux établissements sanitaires et à leurs personnels soignants.
Résumé : Parallèlement à la réforme en cours du système de santé chinois, un financement durable par les pouvoirs publics et des plans de protection sociale adéquats seront indispensables pour assurer l’accès de tous à un traitement adéquat de la tuberculose, afin de réduire le risque de développement de formes multirésistantes et d’organiser systématiquement le traitement et la prise en charge de celles-ci.

Translated from English version into French by Suzanne Assenat, through

TRANSLATORS
WITHOUT BORDERS

Контроль над туберкулезом с лекарственной устойчивостью в Китае: достижения и проблемы

Цянь Лун, Ян Цюй, Генри Лукас

Аннотация

История вопроса. Китай занимает второе место по уровню заболеваемости туберкулезом с множественной лекарственной устойчивостью (ТБ МЛУ) в мире. В 2009 году Правительство Китая приняло решение разработать план по профилактике ТБ МЛУ и борьбе с ним в контексте комплексной реформы системы здравоохранения, начатой в том же году.

Обсуждение. В Китае широко распространен туберкулез с лекарственной устойчивостью и ТБ МЛУ. Заражению ТБ МЛУ намного более подвержены жители бедных сельских районов, а самый высокий уровень заболеваемости фиксируется в слаборазвитых регионах в связи с незаконченным и/или неправильным лечением туберкулеза. Семьи, в которых живут зараженные люди, несут большие финансовые затраты на лечение и связанные с ним расходы. Авторитетная программа по контролю над ТБ МЛУ в Китае, претворяемая в жизнь Глобальным Фондом, обеспечивает техническую и финансовую поддержку диагностированию и лечению ТБ МЛУ. Однако, эта программа действует ограниченное время и не может помочь в долгосрочной перспективе. В 2009 году Фонд Билла и Мелинды Гейтс и Национальная комиссия по вопросам здравоохранения и планирования семьи Китая приступили к разработке инновационных подходов к контролю над туберкулезом/ТБ МЛУ, индивидуальных схем оплаты лечения и увеличению страховых выплат в связи с болезнью пациентов. Такие меры призваны сократить медицинские расходы и устранить финансовые преграды на пути к лечению. Представляется, что эти усилия прилагаются в нужном направлении, но они достигнут цели только при следующих условиях: (а) мобилизации внутренних ресурсов для сбора средств на профилактику туберкулеза/ТБ МЛУ и контроля над заболеванием; (б) должном стимулировании учреждений здравоохранения и медицинского персонала.

Резюме. Наряду с продолжающейся в Китае реформой системы здравоохранения решающее значение для обеспечения всеобщего доступа к лечению от туберкулеза будет иметь стабильное государственное финансирование и медико-социальная защита. В результате необходимо снизить риск заболеваемости ТБ МЛУ, обеспечить систематический контроль над заболеванием и его лечение.

Translated from English version into Russian by Andriy Lapin, through

TRANSLATORS
WITHOUT BORDERS

Control de la tuberculosis resistente a medicamentos en China: progreso y desafíos

Qian Long, Yan Qu, Henry Lucas

Resumen

Antecedentes: China está en segundo lugar a nivel mundial en cantidad de casos de tuberculosis resistente a múltiples drogas (MDR-TB). En el año 2009, el gobierno chino aceptó diseñar un plan para la prevención y control de la MDR-TB en el contexto de una reforma integral del sistema de salud que se lanzara ese mismo año.

Discusión: China está haciendo frente a tasas elevadas de prevalencia de tuberculosis resistente a medicamentos y MDR-TB. La MDR-TB afecta desproporcionadamente a las poblaciones rurales pobres y los índices más elevados se observan en las regiones menos desarrolladas debido principalmente a que los tratamientos para la TB son inadecuados y/o interrumpidos. La mayoría de las familias con un miembro afectado sufren de una importante carga económica debido a la combinación del tratamiento con otros costos asociados. El influyente programa Global Fund para el control de la MDR-TB en China ofrece soporte técnico y financiero para el diagnóstico y tratamiento de la MDR-TB. Sin embargo, este programa tiene un cronograma pre-establecido y no puede ofrecer soluciones a largo plazo. En el año 2009, la Bill and Melinda Gates Foundation, en colaboración con la Comisión de Salud Nacional y Planificación Familiar de China, comenzaron a desarrollar métodos innovadores para el manejo de la TB/MDR-TB y mecanismos de pago basados en cada caso para el tratamiento, junto con un aumento de los beneficios de cobertura de salud para los pacientes, con el fin de contener los gastos médicos y disminuir las barreras financieras para el tratamiento. Si bien estos esfuerzos parecen estar encaminados en la dirección adecuada, es posible que no sean suficientes a menos que (a) se movilicen fuentes domésticas para recaudar fondos para la prevención y control de la TB/MDR-TB y (b) se otorguen incentivos adecuados tanto a instituciones de salud como a los proveedores de asistencia médica.

Resumen: Junto con la actual reforma al sistema de salud en China, el financiamiento sostenido por parte del gobierno y los esquemas de protección de salud social serán de vital importancia para asegurar el acceso universal a los tratamientos adecuados para la TB con el fin de disminuir el riesgo de desarrollo de MDR-TB y el tratamiento y manejo sistemático de la MDR-TB.

Translated from English version into Spanish by Maria Alejandra Aguada, through

TRANSLATORS
WITHOUT BORDERS
